# Determination of *In Vitro* Antimicrobial Activity of Five Sri Lankan Medicinal Plants against Selected Human Pathogenic Bacteria

**DOI:** 10.1155/2019/7431439

**Published:** 2019-05-06

**Authors:** Manikkuwadura Hasara Nethmini De Zoysa, Hasanga Rathnayake, Ruwani Punyakanthi Hewawasam, Weerasinghe Mudiyanselage Dilip Gaya Bandara Wijayaratne

**Affiliations:** ^1^Department of Medical Laboratory Science, Faculty of Allied Health Sciences, University of Ruhuna, Galle, Sri Lanka; ^2^Department of Biochemistry, Faculty of Medicine, University of Ruhuna, Galle, Sri Lanka; ^3^Department of Microbiology, Faculty of Medicine, University of Ruhuna, Galle, Sri Lanka

## Abstract

**Introduction:**

Antibiotic resistance is one of the greatest threats of the 21^st^ century. Scientists search for potential antimicrobial sources that can cope with antibiotic resistance. Plants used in traditional medicine can be identified as potential candidates for the synthesis of novel drug compounds to act against antibiotic-resistant bacteria.

**Objective:**

To determine the potential antimicrobial effects of ethanol, aqueous, and hexane extracts of five Sri Lankan medicinal plants against four human pathogens.

**Methods:**

*Asparagus falcatus* (tubers), *Asteracantha longifolia* (whole plant), *Vetiveria zizanioides* (roots), *Epaltes divaricata* (whole plant), and *Coriandrum sativum* (seeds) were used in the study. Plant extracts were screened against four clinically important Gram-positive and Gram-negative bacterial strains, *Staphylococcus aureus* (ATCC 25923), *Escherichia coli* (ATCC 25922), *Pseudomonas aeruginosa* (ATCC 27853), and *Klebsiella pneumoniae* (ATCC 700603). Antibacterial activity of plant extracts were monitored using the agar disc diffusion method. Eight concentrations of each positive plant extract were used to determine the minimum inhibitory concentration (MIC) by 5-fold dilution of plant extracts yielding a serial dilution of the original extract.

**Results:**

Ethanol, aqueous, and hexane extracts of *E. divaricata* gave the maximum zones of inhibition of 16.3 mm, 7.4 mm, and 13.7 mm and MIC values of 0.48 mg/ml, 1.2 mg/ml, and 1.6 mg/ml, respectively, against *S. aureus*. Ethanol and hexane extracts of *V. zizanioides* gave the maximum zones of inhibition of 12.1 mm and 11.4 mm and MIC values 2.4 mg/ml and 0.003 mg/ml, respectively, against *S. aureus*. None of the other plants were effective against any microorganism used for the study.

**Conclusions:**

It can be concluded that *E. divaricata* and *V. zizanioides* crude ethanol, aqueous, and hexane extracts exhibited significant *in vitro* antibacterial activity against *S. aureus*, and the active compounds isolated from them can be potential sources for the synthesis of antibacterial drugs.

## 1. Introduction

Infectious diseases have increased to a great extent during the recent years [1], and they are the second leading cause of death across the world and the third leading cause of death of economically developed countries [2].

In the recent past, a lot of evidence emerged that human pathogenic microorganisms have developed antibiotic resistance [1]. The existence of microbial strains with reduced susceptibility to antibiotics and increased number of antibiotic-resistant bacterial strains can be caused by indiscriminate use of broad-spectrum antibiotics, immunosuppressive agents, intravenous catheters, organ transplantation, and ongoing epidemic of human immunodeficiency virus (HIV) infections [3]. According to estimations in the USA, 2.22 million hospitalized patients had adverse drug reactions and 106,000 patients died in a single year [4]. Due to multidrug-resistant microbial strains and the adverse effects associated with the synthetic antimicrobial drugs, the scientists search for potential antimicrobial substances from various sources that can combat the issues associated with them [3, 5].

Microorganisms, fungi, algae, symbiotic lichens and mosses, and higher plants are used to develop antimicrobial agents with novel mechanism of action against microorganisms [6]. Throughout the world, medicinal plants and their products have been used in traditional medicine for centuries. Since the beginning of human civilization, plants and plant products are used as medicines [4]. Due to the plethora of evidence documented, medicinal plants would be the best source to obtain a variety of active constituents to be used as antimicrobial agents [3]. Even today, up to 80% of the population in the world depends on ethnomedicine for their medicinal purposes [7]. Sri Lanka is rich in all the three levels of biodiversity, namely, species diversity, genetic diversity, and habitat diversity. Out of the 3,300 flowering plant species in Sri Lanka, 830 (25%) species are endemic to the island [6]. Plant-derived medicines afford benefits such as profound therapeutic benefits, less or no side effects, low cost, and easy accessibility [1, 3].

In the current study, five Sri Lankan traditional medicinal plants were tested for *in vitro* antimicrobial activity against Gram-positive and Gram-negative pathogenic bacteria (Table 1). *Asparagus falcatus* has been used as a cure for tuberculosis, sore throat, and as an antiemetic [7]*. Asteracantha longifolia* has been assayed for antimicrobial activity [8]. *Coriandrum sativum* is not only used in ayurvedic medicine but also important in Aboriginal medicine [9]. *Epaltes divaricata* is a seasonal medicinal plant and has been used to alleviate jaundice, urethral discharge, and acute dyspepsia [10]. *Vetiveria zizinioides* has been used as a cure for rheumatism and malarial fever and as an anthelminthic [2]. All five plants are used by traditional ayurvedic practitioners to alleviate different types of infectious diseases. It was hoped that this research will be beneficial to identify the plant/plants out of the above five plants that would be beneficial to act against the pathogenic bacteria.

Since traditional medicine is growing rapidly, subsequent demand for evidence for the quality, safety, and efficacy of traditional medical services and products has ensued. The scientific validation and creation of research data pertaining to quality, safety, and efficacy of Sri Lankan traditional medicine has become a pressing issue. Such scientific knowledge can help create an evidence-based traditional medicine that is increasingly respected by other public health professionals. Presence of phytochemicals, in addition to vitamins/provitamins and minerals, in fruits and vegetables has recently been considered of crucial nutritional importance in the prevention of chronic diseases [11]. Thus, a complex mixture of phytochemicals in plants provides a better protective effect on health than a single phytochemical. Saponins, tannins, polyphenols, alkaloids, flavonoids, anthraquinones, glycosides, and reducing sugars are some of the widely known phytochemicals identified in medicinal plants. Plants used in this study were not subjected to a complete phytochemical screening so far. Therefore, this study will close that knowledge gap and will provide a better understanding of the phytoconstituents for their various actions reported.

Thus, the objective of this study was to identify the main groups of phytochemicals present in the five medicinal plants screened and to evaluate the antimicrobial activity of aqueous, ethanol, and hexane extracts of each medicinal plant against Gram-positive and Gram-negative pathogenic bacteria.

## 2. Materials and Methods

### 2.1. Plant Collection and Authentication

**Figure 1:**
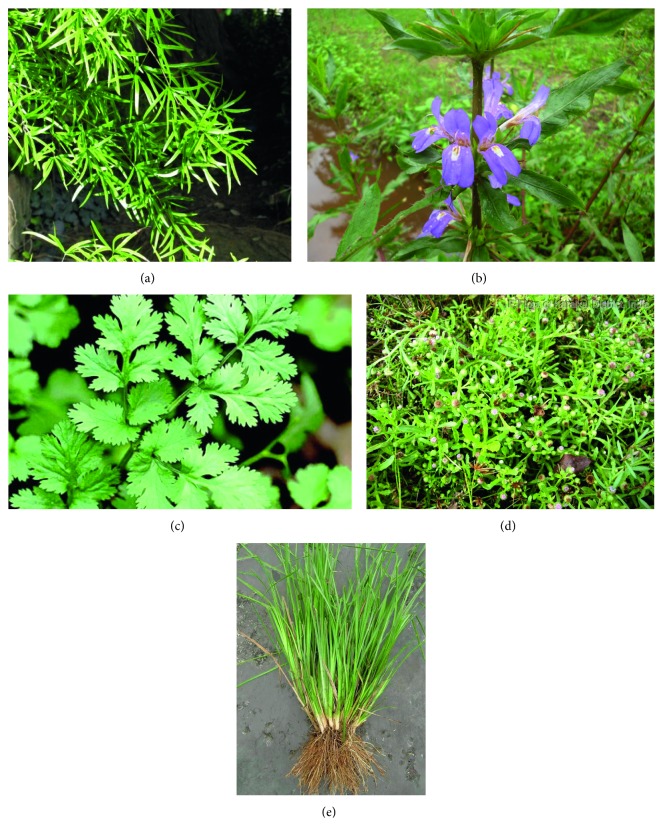
Photographs of plants used in the study. (a) Botanical name: *Asparagus falcatus*; local name: hathawariya; family: Liliaceae. (b) Botanical name: *Asteracantha longifolia;* local name: neeramulliya; family: Acanthaceae. (c) Botanical name: *Coriandrum sativum*; local name: koththamalli; family: Umbelliferae. (d) Botanical name: *Epaltes divaricata*; local name: heen mudamahana; family: Compositae. (e) Botanical name: *Vetiveria zizanioides*; local name: sewendara; family: Graminae.

Fresh plant parts of *Asparagus falcatus*, *Asteracantha longifolia*, *Vetiveria zizanioides*, *Epaltes divaricata*, and *Coriandrum sativum* (Figure 1) were collected from the gardens and villages of Southern Province, Sri Lanka. Plants were authenticated at the National Herbarium, Botanical Gardens, Peradeniya, Sri Lanka. The collected plants were washed with running tap water, air-dried and ground to a coarse powder, and stored in air tight bottles at 4°C.

### 2.2. Phytochemical Assays

#### 2.2.1. Preparation of Plant Extracts

Extracts were prepared according to the respective method used for the analysis of each phytochemical. Unless stated otherwise, all aqueous extracts were prepared by refluxing 2.6 g of powdered dried plant material in 30 ml of distilled water for 1 hr and concentrating the plant extract to a final volume of 20 ml.

#### 2.2.2. Phytochemical Analysis

Major classes of phytochemicals (tannins, alkaloids, phenolic compound, cyanogenic glycosides, cardiac glycosides, reducing sugars, saponins, and flavonoids) were determined by the simple and standard qualitative methods described by Trease and Evans [12] and Sofowora [13]. All methods were optimized with a positive control, and necessary precautions were taken to remove the interference from chlorophyll.

### 2.3. Antimicrobial Assays

#### 2.3.1. Preparation of Crude Extracts

*(1) Solvent Extraction*

- *Ethanol Extract.* Ethanol extracts of all the five medicinal plants were prepared by dissolving 20 g of coarse powder of each plant material separately in 200 ml of ethanol. The contents were kept in a mechanical shaker for 72 hr at 25°C. Then, the extracts were filtered, and the solvent was evaporated using a rotary evaporator. The extracts were resolubilized with 10% DMSO and stored at 4°C in air tight bottles for further studies.
- *Hexane Extract.* 10 g portions of dried plant material were extracted with 100 ml of *n*-hexane using a mechanical shaker for 72 hr at 25°C. Then, the extracts were filtered, and the solvent was evaporated using a rotary evaporator. The extracts were resolubilized with 10% DMSO and stored at 4°C in air tight bottles for further studies.

*(2) Aqueous Extraction*. 2.62 g of dried plant material was refluxed in 60 ml of distilled water for 3 hr. Extract was filtered, evaporated, resolubilized with 10% DMSO, and stored at 4°C in air tight bottles for further studies.

#### 2.3.2. Test Microorganisms

CLSI control strains of *Escherichila coli* (ATCC 25922), *Staphylococcus aureus* (ATCC 25923), *Pseudomonas aeruginosa* (ATCC 27853), and *Klebsiella pneumoniae* (ATCC 700603) were used to check the effectiveness of medicinal plants. Standard bacterial cultures were obtained from Microbiology Department, Faculty of Medicine, University of Ruhuna.

#### 2.3.3. Preparation of McFarland Standard

McFarland number 0.5 standard was prepared by mixing 9.95 ml 1% H_2_SO_4_ in distilled water and 0.05 ml 1% BaCl_2_ in distilled water in order to estimate bacterial density [14]. The preparation was stored in an air tight bottle and used for comparison of bacterial suspension whenever required.

#### 2.3.4. Antimicrobial Susceptibility Assays

*(1) Disk Diffusion Assay*. The antimicrobial susceptibility was initially assayed by the agar disk diffusion method [15]. Three concentrations (crude extract, 10-fold dilution, and 100-fold dilution) of each plant extract were prepared in 10% DMSO. Bacteria cell suspensions were adjusted to 0.5 McFarland turbidity standards to prepare 1 × 10^8^ bacterial/ml inoculum. Each bacterial suspension was inoculated on Mueller-Hinton agar plates, and the plates were then allowed to dry for 5 minutes. The sterile filter paper disks (Whatman No. 1, diameter = 6 mm) were soaked in 10 *μ*l of each plant extract. The extract-soaked filter paper disks were then placed on the inoculated Mueller-Hinton agar plates. Cefotaxime (30 *μ*g) disk was used as the positive control, and 10% DMSO-soaked filter paper disk was used as the negative control. Plates were incubated for 18 hr at 35 ± 2°C. After incubation, the zones of inhibition were recorded as the diameter of the growth-free zones measured in mm using a Vernier caliper.

*(2) Minimum Inhibitory Concentration (MIC)*. Plant extracts that gave a positive result for the disk diffusion assay were used to determine MIC using the microplate dilution method [16]. Serial 5-fold dilutions of the plant extracts were prepared in the 10% DMSO, yielding seven serial dilutions of the original extract. Inoculum of organism was prepared in Mueller-Hinton broth, and the turbidity was adjusted to approximately 0.5 McFarland turbidity standard to prepare 1 × 10^8^ bacterial/ml. 150 *μ*l of plant extract was added to each well of the 96-well microplate. 50 *μ*l of bacterial suspension was added to each well except the negative controls. Cefotaxime IV drug was used as the positive control. 10% DMSO and plant extracts without bacterial suspension were used as the negative controls. Microtiter plates were incubated at 35 ± 2°C for 24 hr. Antimicrobial activity was assessed by measuring absorbance at 630 nm of wave length.

#### 2.3.5. Statistical Analysis

Studies were performed in triplicate. Data were expressed as mean ± SEM.

## 3. Results

### 3.1. Phytochemicals

Out of the main phytochemicals tested, tannins, phenolic compounds, cardiac glycosides, and flavonoids were present in all five plant extracts. Alkaloids were present in all plant extracts except *Asparagus falcatus.* Saponins were present in all other plant extracts except *Coriandrum sativum*. Cyanogenic glycosides and reducing sugars were absent in all five plant extracts (Table 2).

### 3.2. Yield of Extraction

The yield of extraction given by different plant extracts studied is shown in Table 3. Various yields were given by different plant extracts during extraction. Maximum yield was shown by the aqueous extract of *Asparagus falcatus* (34.73%), while the minimum yield was given by the hexane extract of *Asparagus falcatus* (0.5%).

### 3.3. Antimicrobial Activity

#### 3.3.1. Disc Diffusion Assay

**Figure 2:**
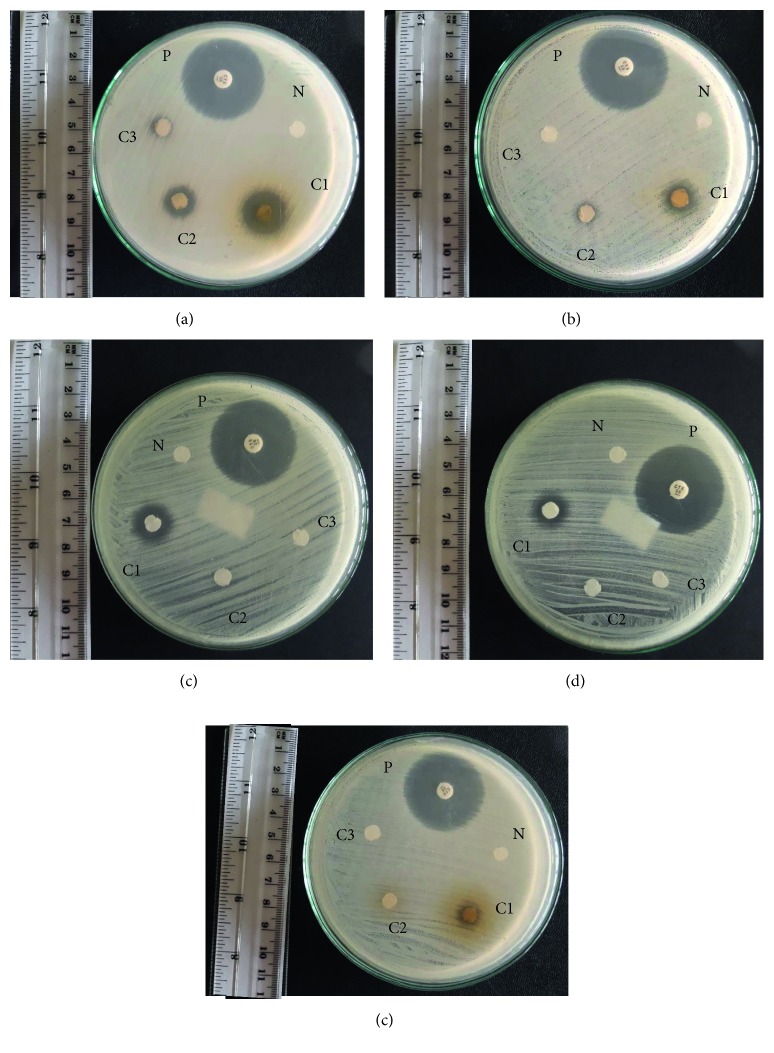
Photographs show only the plants that gave positive results for the disc diffusion assay. (a) Ethanol extract of *E. divaricata*. (b) Ethanol extract of *V. zizanioides*. (c) Hexane extract of *E. divaricata*. (d) Hexane extract of *V. zizanioides*. (e) Aqueous extract of *E. divaricata*. P: positive control; N: negative control; C1: crude extract; C2: 10-fold dilution of crude extract; C3: 100-fold dilution of crude extract.

Among the five plants studied, *Epaltes divaricata* showed a significant inhibition zone in all three extracts (ethanol, aqueous, and hexane) and *Vetiveria zizanioides* showed a significant inhibition zone in two extracts (ethanol and hexane) against *S. aureus* (Figure 2). The concentrations of extracts varied among different plants (Table 4). The antibacterial activities of extracts according to the zone of inhibition ranged between 7.4 and 16.3 mm. Maximum zone of inhibition was observed in the ethanol extract of *Epaltes divaricata* (16.3 mm), and minimum zone of inhibition was given by the aqueous extract of *Epaltes divaricata* (7.4 mm). Aqueous extract of *Epaltes divaricata* gave 7.4 ± 0.2 mm zone of inhibition for crude extract against *S. aureus*. Ethanol extract of *Epaltes divaricata* gave corresponding zones of inhibition 16.3 ± 0.2, 13.4 ± 0.2, and 7.6 ± 0.1 mm, respectively, for crude extract, 10-fold dilution of crude extract, and 100-fold dilution of crude extract while *Vetiveria zizanioides* gave 12.1 ± 0.2 and 7.3 ± 0.3 mm, respectively, for crude extract and 10-fold dilution of crude extract against *S. aureus*. Hexane extract of *Epaltes divaricata* gave 13.7 ± 0.1 mm zone of inhibition for crude extract while *Vetiveria zizanioides* gave 11.4 ± 0.2 mm for crude extract against *S. aureus.* Positive control cefotaxime (30 *μ*g) gave corresponding zones of inhibition 31.2 ± 0.3, 32.9 ± 0.3, 33.7 ± 0.2, and 18.6 ± 0.3 mm, respectively, against *S. aureus*, *E. coli*, *K. pneumoniae*, and *P. aeruginosa*. However, due to the differences in concentrations of extracts, effectiveness of the plant extracts cannot be accurately compared by comparing the respective diameters obtained in the disc diffusion assay. Hence, minimum inhibitory concentration was used to determine the effectiveness of the plant extracts accurately.

#### 3.3.2. Minimum Inhibitory Concentration

The MIC values obtained from plants exhibited antibacterial activity ranged between 0.003 and 2.4 mg/ml (Table 5). Ethanol, aqueous, and hexane extracts of *E. divaricata* gave MIC values of 0.48 mg/ml, 1.2 mg/ml, and 1.6 mg/ml, respectively, against *S. aureus*. Ethanol and hexane extracts of *V. zizanioides* gave MIC values of 2.4 mg/ml and 0.003 mg/ml, respectively, against *S. aureus.* Therefore, the highest antimicrobial activity was observed for the hexane extract of *Vetiveria zizanioides.*

## 4. Discussion

Search for potential antimicrobial drugs increased during the past few decades due to concomitant rise of antibiotic resistant bacterial strains [5]. Studies conducted in several countries have reported that the use of compounds extracted from medicinal plants may be beneficial in the development of antibiotics [17].

There are limited data published on the antimicrobial activity of *Asparagus falcatus*, *Asteracantha longifolia*, *Vetiveria zizanioides*, *Epaltes divaricata*, and *Coriandrum sativum* extracts. But, those reports are also from other countries where the soil composition of the respective region may alter the composition of active compounds and hence the antimicrobial activity present in each plant extract. In the current study, extracts from two plant species gave positive results during the initial antibacterial screening against *S. aureus*. None of the plant extracts were effective against *Escherichila coli, Pseudomonas aeruginosa*, and *Klebsiella pneumoniae.* Ethanol, aqueous, and hexane extracts of *Epaltes divaricata* gave maximum zones of inhibition 16.3 mm, 7.4 mm, and 13.7 mm, respectively, against *S. aureus*. Ethanol and hexane extracts of *Vetiveria zizanioides* gave maximum zones of inhibition 12.1 mm and 11.4 mm, respectively, against *S. aureus*. Zones of inhibition observed with the positive control, cefotaxime, for the organisms used in the study were 31.5 ± 0.2 for *Staphylococcus aureus*, 33.1 ± 0.2 for *Escherichia coli*, and 18.8 ± 0.2 for *Pseudomonas aeruginosa* and were compatible with the published zones of inhibitions such as 25–31 mm, 29–35 mm, and 18–22 mm, respectively.

The MIC was determined against selected bacteria to quantitate the activity of these extracts. *Epaltes divaricata* gave MIC values of 0.48 mg/ml, 1.2 mg/ml, and 1.6 mg/ml, respectively, for ethanol, aqueous, and hexane extracts against *S. aureus*. Ethanol and hexane extracts of *V. zizanioides* gave MIC values of 2.4 mg/ml and 0.003 mg/ml, respectively, against *S. aureus*. According to literature, cefotaxime, the positive control used for this study was given a MIC value between 1 and 4 *μ*g/ml.

*E. divaricata* and *V. zizanioides* have previously been documented as effective against the growth of *S. aureus*, *E. coli*, and *P. aeruginosa* [10]. These differences can be due to the differences in solvents used for extractions in the study. Some of the results of the present study agreed with their findings. However, two species tested in the present study (*E. divaricata* and *V. zizanioides*) exhibited a considerable activity against Gram-positive bacteria. None of the plant species that have been tested in the present study were effective against Gram-negative bacteria.

In a study by Wigmore et al. [5], they have documented that aqueous extracts of plants showed less antibacterial activity than solvent extracts of plants. In the present study also, aqueous extracts showed less antibacterial activity compared to ethanol and hexane extracts. Low polar compounds present in the two active plant extracts may be responsible for this antimicrobial activity.

According to the WHO basic criteria, plants should be nontoxic for the use as therapeutic agents. Five plants used in the study were previously evaluated for subchronic toxicity in mice, and it has already been documented that they are nontoxic in ICR mice [18].

In the present study, *E. divaricata* and *V. zizanioides* have exhibited the antibacterial activity on the Gram-positive bacteria than Gram-negative bacteria. The difference in morphological constituents between Gram-positive and Gram-negative bacteria may be the reason for the differences in antibacterial sensitivity. The structural lipopolysaccharide components in outer phospholipid membrane of Gram-negative bacteria cause the impermeability of cell wall to antimicrobial chemical substances. The Gram-positive bacteria have an outer peptidoglycan layer, which makes the cell wall more permeable to antimicrobial substances than lipopolysaccharide layer. Therefore, the complexity of the cell walls of Gram-negative bacteria is higher than that of Gram-positive bacteria. Therefore, Gram-negative bacteria are less susceptible to antimicrobial chemical substances than Gram-positive bacteria [8].

Compounds extracted during the extraction procedure are mainly dependent on the type of the solvent. Water is the primary solvent used in the traditional medicine, but in the present study, compounds extracted in organic solvents (ethanol and hexane) have exhibited more significant antibacterial activity compared to those extracted in water. Among the extracts investigated, hexane extract exhibited the highest antibacterial effect followed by the ethanol extract. These observations can be due to the polarity of the compounds which were extracted by each solvent and the ability of extracts to diffuse and dissolve in different culture media used in the study. Among the three solvents used in the study, water is the most polar solvent and hexane is the least polar solvent. The lowest MIC value was given by *V. zizanioides* hexane extract. This MIC value is almost similar to the MIC value shown by cefotaxime, the positive control used for the study. When consider *E. divaricata*, the ethanol extract exhibited the lowest MIC value. According to the results of the present study, less polar compounds exhibited more antibacterial activity compared to more polar compounds.

Absence of antimicrobial activity does not mean that the bioactive compounds are not present in the plant or the plant has no activity against microorganisms. Presence of inadequate quantities of active constituent or constituents in the extract to exhibit the antimicrobial activity can be the reason for the negative results.

Phytochemical studies have shown that plants with antimicrobial activity contain bioactive constituents such as tannins, flavonoids, alkaloids, and saponins [19]. Polyphenols in plants include flavonoids, phenolic acids, stilbenes, and lignans [20]. Preliminary research indicates that flavonoids may modify allergens, viruses, and carcinogens and so may be biological “response modifiers.” *In vitro* studies show that flavonoids also have antiallergic, anti-inflammatory, antimicrobial, anticancer, and antidiarrheal activities [21]. Saponins seem to stimulate the immune system [22]. Therefore, presence of polyphenolic compounds, alkaloids, tannins, and saponin may contribute to the antimicrobial activity of above plants.

## 5. Conclusion

The results obtained from this study provided evidence that the ethanol and hexane extracts of the Sri Lankan traditional medicinal plants *E. divaricata* and *V. zizanioides* and aqueous extract of *E. divaricata* exhibited beneficial antibacterial activity against *S. aureus* (ATCC 25923). None of the other plant extracts were active against any of the microorganisms tested. The highest antimicrobial activity was observed for hexane extract of *Vetiveria zizanioides* which showed a MIC value in the same range as the positive control used. It can be concluded that a low polar active compound present in the *Vetiveria* extract may be responsible for the significant antimicrobial activity. Many polyphenolic constituents and alkaloids derived from plants are known to have antimicrobial activity. Therefore, presence of these compounds in the above plant extracts may be responsible for their antimicrobial action. Further scientific evaluation of these plants should be done including fractionation and further characterization of phytochemicals to identify the active components responsible for the antimicrobial activity, as well as to adjudge the *in vivo* activities of these constituents.

## Figures and Tables

**Table 1:** Plants used for the study.

Plant botanical name	Family	Local name
*Asparagus falcatus*	Liliaceae	Hathawariya
*Asteracantha longifolia*	Acanthaceae	Neeramulliya
*Coriandrum sativum*	Umbelliferae	Koththamalli
*Epaltes divaricata*	Compositae	Heen mudamahana
*Vetiveria zizanioides*	Graminae	Sewendara

**Table 2:** Summary of the phytochemicals present in five plant species.

Phytochemical	Plant species				
	*A. falcatus*	*A. longifolia*	*V. zizanioides*	*E. divericata*	*C. sativum*
Tannins	+	+	+	+	+
Phenolic compounds	+	+	+	+	+
Reducing sugars	−	−	−	−	−
Saponins	+	+	+	+	−
Alkaloides	−	+	+	+	+
Cyanogenic glycosides	−	−	−	−	−
Cardiac glycosides	+	+	+	+	+
Flavonoids	+	+	+	+	+

Positive mark (+) indicates the presence of the phytochemical. Negative mark (−) indicates the absence of the phytochemical.

**Table 3:** Yield of extraction (%) of different extracts of five plants.

Plant	Parts used	Yield of extraction (%)		
		Aqueous	Ethanol	Hexane
*Asparagus falcatus*	Tubers	34.73	9.60	0.5
*Asteracantha longifolia*	Whole plant	24.81	4.38	3.6
*Coriandrum sativum*	Seeds	9.54	5.33	1.2
*Epaltes divaricata*	Whole plant	22.90	7.74	1.1
*Vetiveria zizanioides*	Roots	9.92	4.03	0.8

**Table 4:** Concentrations of crude extracts of plants.

Medicinal plant	Concentration (mg/mL)		
	Water extract	Ethanol extract	Hexane extract
*Asparagus falcatus*	300	550	25
*Asteracantha longifolia*	210	290	90
*Coriandrum sativum*	125	310	60
*Epaltes divaricata*	150	310	36
*Vetiveria zizanioides*	80	320	40

**Table 5:** MIC values of plant extracts against *S. aureus*.

Plant	MIC (mg/ml)		
	Aqueous extract	Ethanol extract	Hexane extract
*Epaltes divaricata*	1.2	0.48	1.6
*Vetiveria zizanioides*	—	2.4	0.003

## Data Availability

The data used to support the findings of present study are available from the corresponding author upon request.
